# Sex Differences in Facial and Vocal Attractiveness Among College Students in China

**DOI:** 10.3389/fpsyg.2019.01166

**Published:** 2019-05-22

**Authors:** Juan Hou, Zi Ye

**Affiliations:** Department of Philosophy, Anhui University, Hefei, China

**Keywords:** sex differences, facial attractiveness, vocal attractiveness, mate preferences, college students

## Abstract

This study aims to investigate sex differences in ratings for facial attractiveness (FA) and vocal attractiveness (VA). Participants (60 undergraduates in Study 1 and 111 undergraduates in Study 2) rated the attractiveness of computerized face images and voice recordings of men and women. In Study 1, face images and voice recordings were presented separately. Results indicated that men generally rated voice recordings of women more attractive than those of men, whereas women did not show different attractiveness ratings for voices of men vs. women. In Study 2, face images and voice recordings were paired as multimodal stimuli and presented simultaneously. Results indicated that men rated multimodal stimuli of women as more attractive than those of men, whereas women did not differentiate multimodal stimuli of men vs. women. We found that, compared to VA, FA had a stronger influence on participants' overall evaluations. Finally, we tested the difference between “original multimodal stimuli” (OMS) and “non-original multimodal stimuli” (non-OMS) and found the “OMS-facilitating effect.” Taken together, findings indicated some sex differences in FA and VA in the current study, which could be used to interpret behaviors of sexual selection, human mate preferences, and designs and popularization of sex robots.

## Introduction

People show different preferences for certain human physical attractiveness, such as facial attractiveness (FA) and vocal attractiveness (VA). These preferences play significant roles in people's mate preferences across cultures and regions (Buss, 2007). From the theoretical perspective of evolutionary psychology (Barber, 1995), people's tendency to take attractiveness of physical cues is deeply rooted in their psychology as a primary criterion to evaluate potential mates and has been functional for survival and reproduction of the species. According to this theory, FA often provides information about targets' physical health and fertility so that it can indicate signs about the quality of one's genes to some extent (Barber, 1995). Therefore, in the mate processes, human beings instinctively evaluate potential partners' evolutionary fitness, physical health, and prospect for reproduction by detecting their physical cues (e.g., faces, voices, skins, statures, or hairs; Williams, 1975; Symons, 1979).

### FA Under the Framework of Evolutionary Psychology

Over the past few years, evolutionary psychologists have conducted extensive researches on visual cues, such as facial symmetry, facial cleanliness, and sexual-dimorphism-related facial features. Facial symmetry, as a vital criterion of FA and one of the indicators of gene quality, is positively correlated with people's sexual enchantment and physical and psychological health. For instance, people who have symmetrical faces scored higher on tests of physiological, psychological, emotional health (Shackelford and Larsen, 1997), and show the capacity of withstanding pressures from the environment (Little et al., 2011). Regarding facial cleanliness, Johnstone et al. (2001) found that people with smooth and clear skins (without scars) are perceived as more attractive. Moreover, regarding sexual-dimorphism-related facial features, women are inclined to prefer masculine male faces with obvious secondary sexual characteristics, which advertise the quality of male genes (Little et al., 2011).

### VA Under the Framework of Evolutionary Psychology

In recent years, the suggestion that VA plays a vital role in human mating has drawn increasing attention among researchers (Owren, 2011; Wu et al., 2014). Vocal pitches, for example, reflect people's evolutionary fitness, health conditions, and fertility. Women show an increased preference to lower as opposed to higher pitched male voices, whereas men show an increased preference to higher as opposed to lower-pitched female voices (Buss, 2007). Kempe et al. (2013) also supported that men with low and deep voices (masculine voices) secrete more testosterone, which is related to health and a reduced likelihood of illness (Thornhill and Gangestad, 2006).

Concerning women, higher-pitched voices are correlated with their higher WHR (Waist-to-Hip Ratio) and SHR (Shoulder-to-Hip Ratio), which are essential cues of fertility (Buss, 2007). Therefore, high-pitched feminine voices reflect high fertility and evolutionary fitness. Besides, higher-pitched female voices are more attractive to men than lower-pitched voices because higher-pitched voices are more likely to come from younger women with an attractive figure and hormonal profile (Collins and Missing, 2003). To increase VA, women tend to raise their vocal pitch in mate processes either consciously or unconsciously (Fraccaro et al., 2011).

### Sex Differences and Weight of FA and VA

Some sex differences and the effect of “sex facilitation” (EOSF)—behaviors elicited by the preference for opposite-sex stimuli—are found in FA, but have not yet been investigated in VA. Generally, men evaluate female FA as higher than male FA; however, women do not show a similar tendency in evaluation male FA higher than female FA. Moreover, men are more likely to pay attention to beautiful women than handsome men, and they display more risky behaviors after watching women with high FA (Elzinga et al., 2011). Due to EOSF, men show different work performances when women are present; conversely, women are less likely to display distinctive behaviors when men are present (Shan et al., 2010). However, there are relatively few studies focusing on sex differences in VA, and the EOSF found in FA has not yet been replicated in VA. Thus, it remains unknown and is worth investigating whether female voices can elicit male attention or enable them to take more risks in similar ways as female faces.

Both FA and VA reflect people's health conditions and fertility, which are related to gene quality or “evolutionary fitness.” Then, are perceived FA and VA probably consistent with each other because they are both from the gene? Indeed, Hill et al. (2017) found that facial asymmetry (an indicator of FA; *low* facial asymmetry suggests *high* attractiveness) is negatively correlated with VA, even though the coefficient *r*s were relatively low, ranging from −0.10 to −0.36. In other words, people who have more attractive faces are more likely to possess attractive voices to some extent. Evidence also supported that same-individual FA and VA share concordant evaluations regarding the degree of femininity or masculinity, conditions of health, etc. (Smith et al., 2016). We expected, therefore, that same-individual FA and VA would be correlated since both FA and VA reflect people's health conditions or fertility, which are related to gene quality or “evolutionary fitness.”

Compared to VA, FA plays a more vital role in perception of integrated impression (overall attractiveness). Stevenage and Neil (2014) suggested that the auditory channel is a separated but parallel pathway in the process of human perception, despite its relatively weaker functioning compared to the visual channel. Rezlescu et al. (2015) also supported that people evaluated faces as more attractive than voices in human mating. Furthermore, the influence of FA is nearly triple that of VA when evaluating competence (Klofstad, 2017). For sex differences, Puts et al. (2014) theorized that VA of men and women is valued differently by different sex in the mate processes. In particular, male voices are more valued than female voices, suggesting that male voices provide more information about people's physiological status than female voices (Puts et al., 2014). However, this theory has not been tested empirically. Thus, the weight of FA and VA in integral impression (overall attractiveness) are worthy to investigate because it is helpful to extract separated effects and functioning of FA and VA, as well as revealing its evolutionary meaning in human mating. We expected, therefore, that the predictive effects (weight) of FA and VA to the integrated impression (overall attractiveness) would be distinct.

### Facilitating Effect of Original Multimodal Stimuli (OMS-Facilitating Effect)

Differences and weight of FA and VA can be investigated by combining facial and vocal stimuli and simultaneously presenting them. However, same-individual faces and voices (i.e., the face and voice belonging to the same individual) may share some common traits that facilitate people to perceive them as more attractive relative to different-individual ones (i.e., the face and voice belong to different people). According to Smith et al. (2016), people generally have more than 50% chance of making correct judgments on whether a multimodal stimulus (faces and voices) belongs to the same person. Moreover, multimodal stimuli combined with same-individual faces and voices are more attractive than those combined with different-individual faces and voices (Stevenage and Neil, 2014). Based on these findings, we predicted that “original multimodal stimuli (OMS)” (multimodal stimuli consist of same-individual faces and voices) would be evaluated as more attractive than “non-original multimodal stimuli (non-OMS)” (multimodal stimuli consist of different-individual faces and voices)—we call this phenomenon as “OMS-facilitating effect.”

### Overview and Hypothesis of the Current Study

Building upon the above-reviewed theories and research, the present study tested sex differences in preferences for male and female faces and voices. Specifically, we tested whether men and women differ in their preferences for faces and voices of the same vs. the opposite sex. Moreover, we tested whether perceptions of FA and VA vary in sex (raters' sex and stimuli's sex), the mode of stimuli (faces vs. voices) or their interactions. Finally, we tested the difference between OMS and non-OMS to investigate the “OMS-facilitating effect.”

We expected to find some differences of perceived FA and VA between men and women and differences in separately predictive effects of FA and VA (attractiveness score in Study (1) to multimodal stimuli (overall attractiveness score in Study (2) by conducting two related studies among Chinese college students. First, we conducted Study 1 to test: Hypothesis (1), People with attractive faces would be rated as having attractive voices. Hypothesis (2), Although both male and female raters could consistently give attractive faces and voices higher ratings (2a), men would evaluate opposite-sex FA or VA as more attractive than same-sex ones (2b); the EOSF would not be significant among female raters (2c). Next, we conducted Study 2 to test: Hypothesis (3), faces would have a stronger impact (weight) on attractiveness than voices in general; Hypothesis (4), OMS would be evaluated as more attractive than non-OMS.

## Study 1

To generate facial and vocal stimuli, we recruited participants to collect their facial photographs and vocal recordings. Then, we recruited other participants as raters to evaluate FA and VA of these stimuli. We tested correlations between same-individual FA and VA (Hypothesis 1), the consistency of different-sex evaluation on FA and VA (Hypothesis 2a) and EOSF among male raters (Hypothesis 2b) rather than among female raters (Hypothesis 2c) in this stage.

### Methods

#### Participants

Sixty-five college students from Anhui University participated in Study 1. Students reported their sexual orientation with a scale of self-evaluation of homosexuality tendency—“*Please evaluate your homosexuality tendency from 1 (completely homosexual tendency) to 9 (no homosexual tendency at all)*.” We excluded data from participants whose score of homosexuality tendency reached or exceeded 5 (Wang et al., 2015). Sixty participants remained (30 men, *M*_age_ = 20.90, *SD* = 1.37); 30 women, *M*_age_ = 20.47, *SD* = 1.26), which yields over 90% statistical power to find an effect (*f* ^2^ = 0.22). Participants were thanked and received a gift after completing the experiment.

#### Ethics Statement

The study was approved by the Human Research Ethics Committee of Anhui University. All participants gave consent to participate in the study and principles expressed in the Declaration of Helsinki were closely followed. Participants were undergraduate students. The youngest participant was 18 years old. Informed consent was obtained in written form from all participants. These college students were considered to have comparable intelligence and ability, and able to take charge of their behaviors.

#### Materials

**Figure 1:**
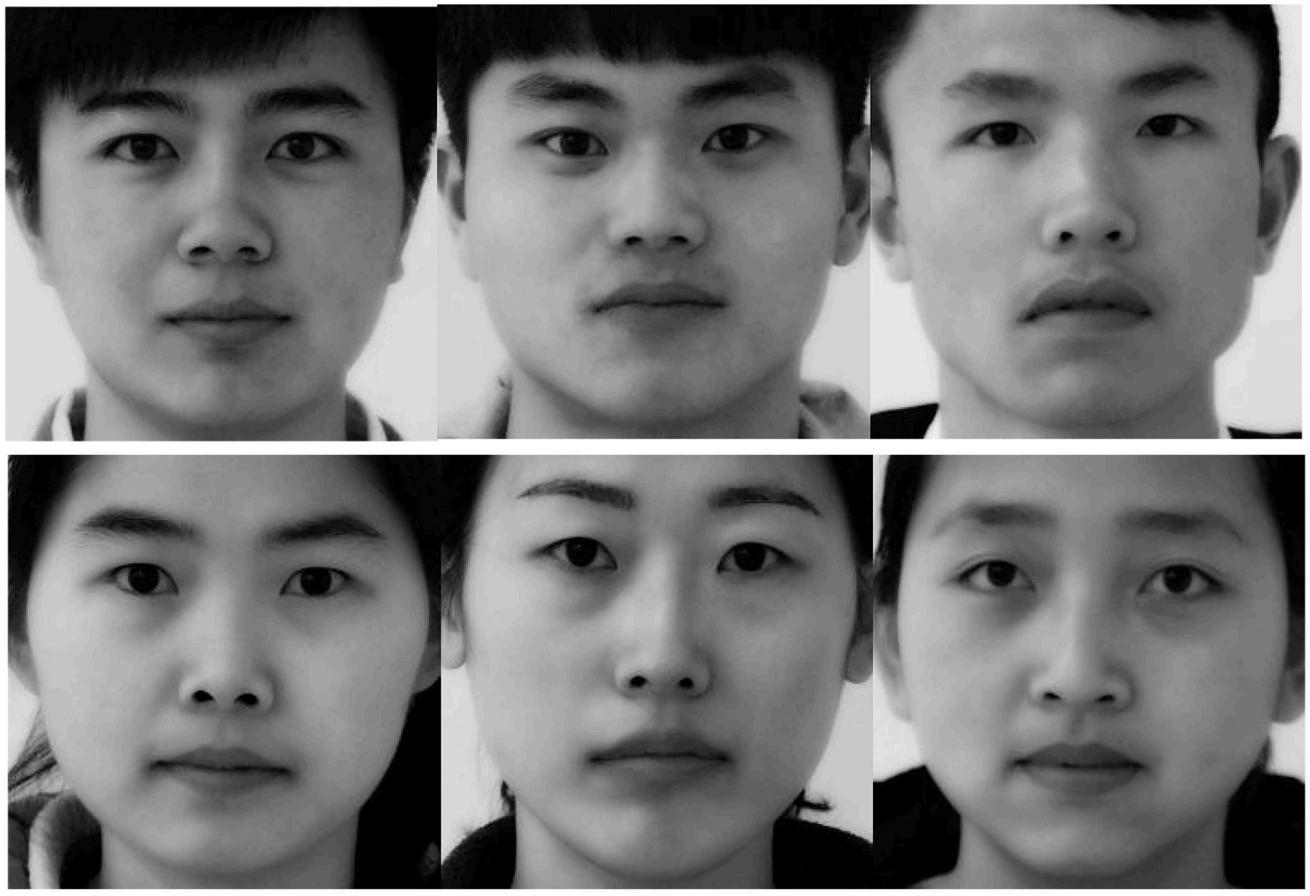
Examples: photographs collected and used into Study 1 and Study 2 (high-, average- and low-attractive level from left to right). Participants have signed written informed consent for the publication of their face photographs.

Twenty college students from Anhui University, including ten men and ten women aged between 17 and 23 (*M*_age_ = 21.3, *SD* = 1.19), provided their faces and voices as experimental stimuli. They were all healthy, without scars or surgical history on faces and throats and familial-hereditary diseases. All students signed written informed consent and allowed their face photographs and voice recordings to be used in this study and for publication. Examples of facial photographs see Figure 1.

We collected facial photographs and vocal recordings of these students. We photographed students with SLR (single lens reflex) camera for their faces. Afterward, all participants listened to the example of “good morning” (in Chinese) in both male and female versions before saying “good morning” with a flat tone. Participants could get a gift as a reward after collecting experimental materials.

Then, we entered and coded these materials into computers. Facial photographs were modified into black and white (unify grayscale), uniform sizes, resolution, and sensitometer with Adobe Photoshop CS6 and Adobe Lightroom. We denoised and unified the duration (3,000 ms) and loudness of vocal recordings. Finally, we ruled out two pairs of stimuli (one male and one female) due to their loud noise. Eighteen pairs of stimuli (nine male and nine female pairs; a pair of stimulus consists of one face and one voice) remained for later experiments. Images and recordings were separately presented as facial and vocal stimuli in computers with E-prime.

#### Procedure

**Figure 2:**
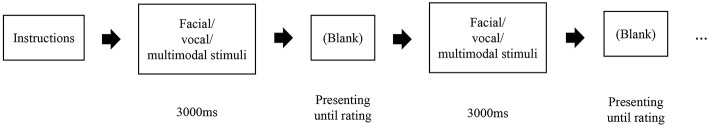
The procedure of presenting facial/vocal stimuli in Study 1 and multimodal stimuli in Study 2.

All participants received all 18 facial and 18 vocal stimuli separately. Participants were asked to read the 18 facial images (section Introduction) and listen to the 18 vocal recordings (section Study 1) and rated each stimulus on 9-point scales (*1* = “*very unattractive*,” *9* = “*very attractive*”). The procedure of presenting stimuli shows in Figure 2.

In section Introduction, participants saw facial photographs in the computer and evaluated FA of the stimuli from 1 to 9. Each stimulus lasted for 3,000 ms and was randomly presented twice.

In section Study 1, the same as in section Introduction, participants listened to recordings from headphones and evaluated VA of the stimuli from 1 to 9. Each stimulus lasted for 3,000 ms and was randomly presented twice.

#### Statistical Analyses

First, we evaluated the test-retest reliability with Pearson correlation (*r*s) in repeating ratings of each stimulus. Next, we evaluated the inter-rater reliability amongst raters with Intraclass Correlation Coefficient (ICCs). Then, we used Spearman correlation to investigate the relationship between ranks of same-individual faces and voices. Finally, we employed MANOVA to test sex-dependent effects both in raters and stimuli.

### Results

First, we tested the correlation of repeating evaluations on stimuli (each stimulus was evaluated twice by one rater) in order to investigate the test-retest reliability. Repeating scores of both facial stimuli (minimum *r* > 0.69, *p* < 0.01) and vocal stimuli (minimum *r* > 0.51, *p* < 0.01) were significantly correlated (correlation coefficients refer to Table S1).

We evaluated the inter-rater reliability by looking at the ICCs in ratings of faces and voices amongst different raters. Results show high inter-rater reliabilities in facial and vocal ratings, ICCs = 0.975, *p* < 0.0001.

Then, we employed Spearman Correlation, due to the limited number of stimuli (9 pairs), to test attractiveness of facial and vocal stimuli in order to investigate whether people with attractive faces are more likely to be rated as having attractive voices (Hypothesis 1). Results displayed that correlation coefficient (rho) was −0.009, which was insignificant. Score and ranks of FA and VA refer to Table S2.

**Figure 3:**
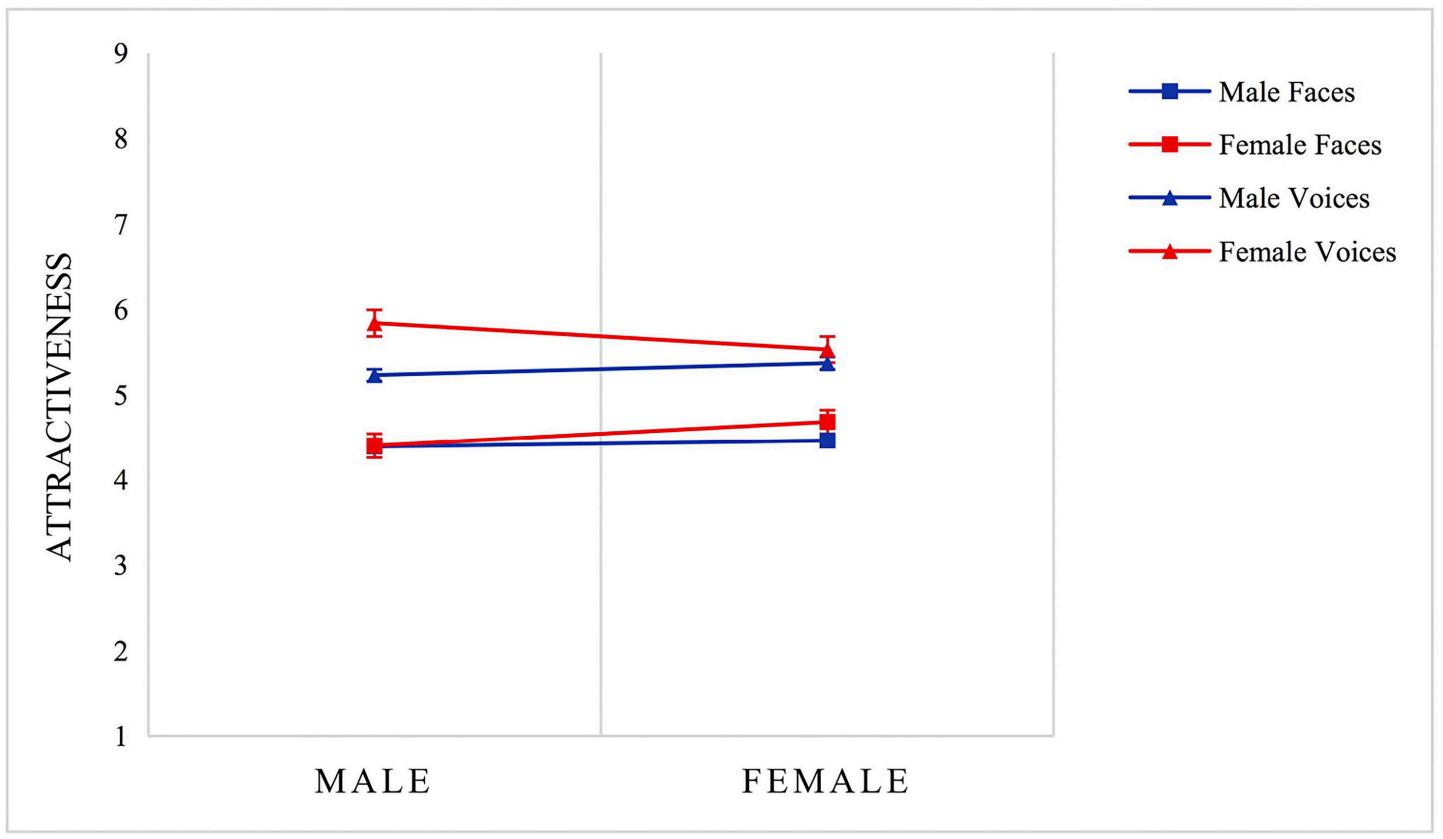
MANOVA analysis of attractiveness of different-sex stimuli × different-sex raters in Study 1. Error bars indicate standard errors of the mean.

Then, we tested effects of raters' sex and sex of facial and vocal stimuli with MANOVA - 2 (raters' sex: male, female) × 2 (facial stimuli' sex: male, female) and 2 (raters' sex: male, female) × 2 (vocal stimuli's sex: male, female). It is noticeable in Figure 3 that the main effect of vocal stimuli's sex was significant [*F*_(1, 58)_ = 15.31, *p* < 0.01, $\eta_{p}^{2}$ = *0.2*1]; however, we did not find any significant effect of raters' sex. We also found significant interactive effects between these two factors [*F*_(1, 58)_ = 5.00, *p* < 0.05, $\eta_{p}^{2}$ = 0.08]. Specifically, male raters evaluated female voices (*M*_score_ = 5.84, *SD* = 1.22) significantly more attractive than male voices (*M*_score_ = 5.23, *SD* = 1.26) [*F*_(1, 58)_ = 18.91, *p* < 0.01]. Similar effects, however, were not found among female raters. On the other hand, there was a subtle difference in facial stimuli. For descriptives see Table 1.

**Table 1:** Mean, SD, and MANOVA of raters' sex and stimuli's sex in Study 1.

	**Attractiveness**						
	**Stimuli's sex**	**Raters' sex**	***M* (*SD*)**	**Predictors**	***df***	***F***	***Partial η^2^***
Facial stimuli	Male	Male	4.39 (1.35)	Raters' sex	58	0.32	0.00
		Female	4.40 (1.18)				
	Female	Male	4.46 (1.27)	Stimuli's sex	58	1.11	0.02
		Female	4.68 (1.25)				
	—	—	—	Raters' sex × stimuli's sex	58	0.94	0.02
	—	—	—				
Vocal stimuli	Male	Male	5.23 (1.26)	Raters' sex	58	0.08	0.00
		Female	5.84 (1.22)				
	Female	Male	5.37 (1.21)	Stimuli's sex	58	15.31^**^	0.21
		Female	5.53 (1.25)				
	—	—	—	Raters' sex × stimuli's sex	58	5.00^*^	0.08
	—	—	—				

* p < 0.05,

** *p < 0.01*.

### Discussion

The positive correlations of two evaluations on repeating stimuli indicate that both facial and vocal stimuli were reliable and valid, and raters could evaluate them consistently. Therefore, these stimuli's perceived (subjective) attractiveness score could reasonably be regarded as their nature or attributes (objective attractiveness score). Given that, it would be plausible to use the score of FA and VA generated in Study 1 to predict perceived attractiveness score of multimodal stimuli in Study 2.

Results of Spearman Correlation, however, were insignificant, suggesting that FA and VA of stimuli were independent rather than having a positive relationship as we had assumed in Hypothesis (1). The insignificance is probably because of the limited number of stimuli, which made a positive correlation between individuals' FA and VA not robust. Otherwise, the absence of positive same-individual FA-VA correlations actually may also account for the lack of significance.

As we predicted in Hypothesis (2a), the insignificance of raters' sex suggests that different-sex participants were able to perceive and evaluate FA and VA consistently. This ability may result from people's consistent psychological criteria to both same- and opposite-sex physical attractiveness. Interestingly, we only found EOSF among male raters, which supported Hypothesis (2b) that men would evaluate opposite-sex VA as more attractive than same-sex VA; however, we did not find EOSF among female raters, which supported Hypothesis (2c). Nevertheless, we just revealed these effects through separated stimuli (either facial or vocal stimuli). To make the results more practical and reliable, we could further test effects through multimodal stimuli (pairs consisting of faces and voices).

## Study 2

To replicate consistency of evaluation and EOSF found in Study 1, we tested the predictive effects of FA and VA (Hypothesis 3) and “OMS-facilitating effect” (Hypothesis 4) in multimodal stimuli. We selected and paired facial and vocal stimuli in Study 1 to generate multimodal stimuli, which would be simultaneously presented and rated as a whole. As discussed, we regarded the scores of facial and vocal stimuli in Study 1 as their nature and used them to predict the perceived attractiveness of multimodal stimuli in Study 2. Based on the results of Study 1, we expected that different-sex raters could evaluate FA, VA, and the attractiveness of multimodal stimuli consistently. Moreover, we expected the EOSF only among male raters. We also expected that FA would predict to more extent in the attractiveness of multimodal stimuli than VA because the visual channel takes more weight in people information inputs than the auditory channel.

### Methods

#### Participants

One hundred and forty-four college students were recruited from Anhui University. The participants who had participated in Study 1 were not recruited to participate in Study 2 any longer. Participants filled out the same scale of self-evaluation of homosexuality tendency as in Study 1. One hundred and eleven students remained (50 men, *M*_age_ = 20.78, *SD* = 1.64); 61 women, *M*_age_ = 20.73, *SD* = 1.33), which yields over 90% statistical power to find predicted effect (*f* ^2^ = 0.12). Participants willingly participated in this experiment and signed informed consent, as well receiving a gift as a reward for taking part.

#### Ethics Statement

The study was approved by the Human Research Ethics Committee of Anhui University. All participants gave consent to participate in the study and principles expressed in the Declaration of Helsinki were closely followed. Participants were undergraduate students. We did not obtain informed consent from the next of kin, caretakers, or guardians on behalf of the minors/children enrolled in our study. Informed consent was obtained in written form from all participants.

Only one participant was 17 years old. We did not obtain consent from his guardians. This young college student was considered to have comparable intelligence and ability to adult students, and able to take charge of his behavior. According to the General principles of the Civil Law of the People's Republic of China; “A minor aged 10 or over shall be a person with limited capacity for civil conduct and may engage in civil activities appropriate to his age and intellect; in other civic activities, he shall be represented by his agent ad litem or participate with the consent of his agent ad litem” (Article 12, Chapter II). Therefore, we obtained the same consent from this participant as those above 18 years old, which was also approved by the Human Research Ethics Committee of Anhui University.

#### Materials

To make the attractiveness of stimuli highly distinguishable from each other and strongly represent different levels of attractiveness, we selected stimuli (used in Study 1) based on their attractiveness. Among *male facial images*, for example, we selected six (two top attractiveness scores, two bottom attractiveness scores and two closest to the mean attractiveness score) from nine *male faces* to represent high-, average- and low-attractiveness facial stimuli. The same approach was employed to select female facial stimuli and all vocal stimuli (examples of facial stimuli see Figure 1). As a result, we have elected six *male faces*, six *female faces*, six *male voices*, and six *female voices*. Afterward, we paired facial stimuli with same-sex vocal stimuli as multimodal stimuli (each multimodal stimulus consists of a facial and a vocal stimulus). As a result, we generated 72 pairs of multimodal stimuli (36 male and 36 female pairs; a pair of multimodal stimuli consists of one face and one voice), including nine original multimodal stimuli (OMS) which consist of same-individual facial and vocal stimuli. However, some stimuli could not be paired as OMS resulting from the rule—selecting six out of nine stimuli. For example, a man's face was selected and his voice was ruled out. To generate as many OMS as possible, we paired his face elect with his ruled-out voice as an OMS, adding them into the stimuli pool. As a result, we had 78 pairs of stimuli in total in Study 2.

#### Procedure

All participants received all 78 pairs of stimuli. Participants simultaneously saw and listened to multimodal stimuli, which were randomly presented, in the computer and from the headphone. Meanwhile, participants evaluated attractiveness of these multimodal stimuli on a 9-point scale (*1* = “*very unattractive*,” *9* = “*very attractive*”). Each stimulus lasted for 3,000 ms and was randomly presented twice. The procedure of presenting stimuli shows in Figure 2.

#### Statistical Analyses

Similar to Study 1, we evaluated the test-retest reliability with Pearson correlation in repeating ratings of each stimulus. Next, we evaluated the inter-rater reliability amongst raters with ICC. Then, we employed MANOVA to test sex-dependent effects both in raters and stimuli, using $\eta_{p}^{2}$ to indicate effect sizes. Then, we employed multiple regressions to investigate the predictive effects of facial and vocal stimuli to multimodal stimuli. Finally, we employed paired-sample *t*-test to test the effect between OMS and non-OMS in order to find the OMS-facilitating effect.

### Results

First, repeating ratings of each multimodal stimulus were significantly correlated with each other (*n* = 111, each of *r* > 0.37, *p* < 0.01). Therefore, all multimodal stimuli had relatively high reliability (correlation coefficients refer to Table S3).

Then, results show a high inter-rater reliability for multimodal stimuli amongst raters, ICC = 0.932, *p* < 0.001.

**Figure 4:**
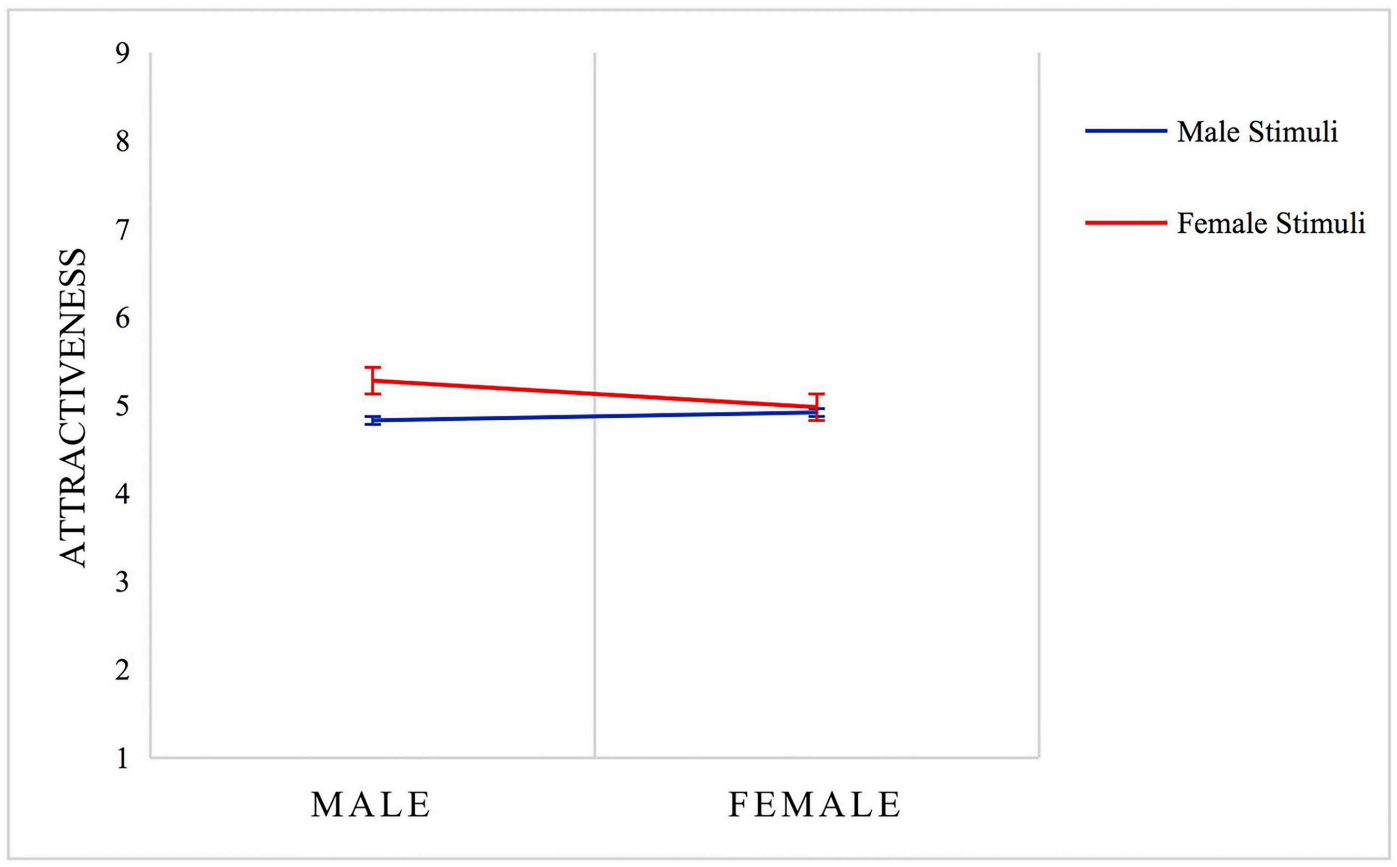
MANOVA analysis of attractiveness of different-sex multimodal stimuli × different-sex raters in Study 2. Error bars indicate standard errors of the mean.

We employed MANOVA to test the effects of stimuli's sex and raters' sex−2 (raters' sex: male, female) × 2 (stimuli's sex: male, female). The results showed that the main effect of stimuli's sex was significant [*F*_(1, 109)_ = 12.89, *p* < 0.01, $\eta_{p}^{2}$ = 0.11]; however, the main effect of raters' sex was insignificant. The interactive effect between these two factors was significant as well [*F*_(1, 109)_ = 7.17, *p* < 0.01, $\eta_{p}^{2}$ = 0.06] (Figure 4). In particular, male raters evaluated female stimuli as more attractive (*M* = 5.28, *SD* = 0.97) than male stimuli (*M* = 4.83, *SD* = 0.99) [*F*_(1, 109)_ = 18.17, *p* < 0.01]; the similar effect, however, was not found among female raters (Descriptive results see Table 2).

**Table 2:** Mean, SD, and MANOVA of raters' sex and multimodal stimuli's sex in Study 2.

**Attractiveness**						
**Rater's sex**	**Stimuli's sex**	***M (SD)***	**Predictors**	***df***	***F***	***Partial η*^2^**
Male	Male	4.83 (0.99)	Raters' sex	109	0.36	0.00
	Female	5.28 (0.97)				
Female	Male	4.92 (0.97)	Stimuli's sex	109	12.89^**^	0.11
	Female	4.98 (1.08)				
—	—	—	Raters' sex × stimuli's sex	109	7.17^**^	0.06
—	—	—				

** *p < 0.01*.

**Figure 5:**
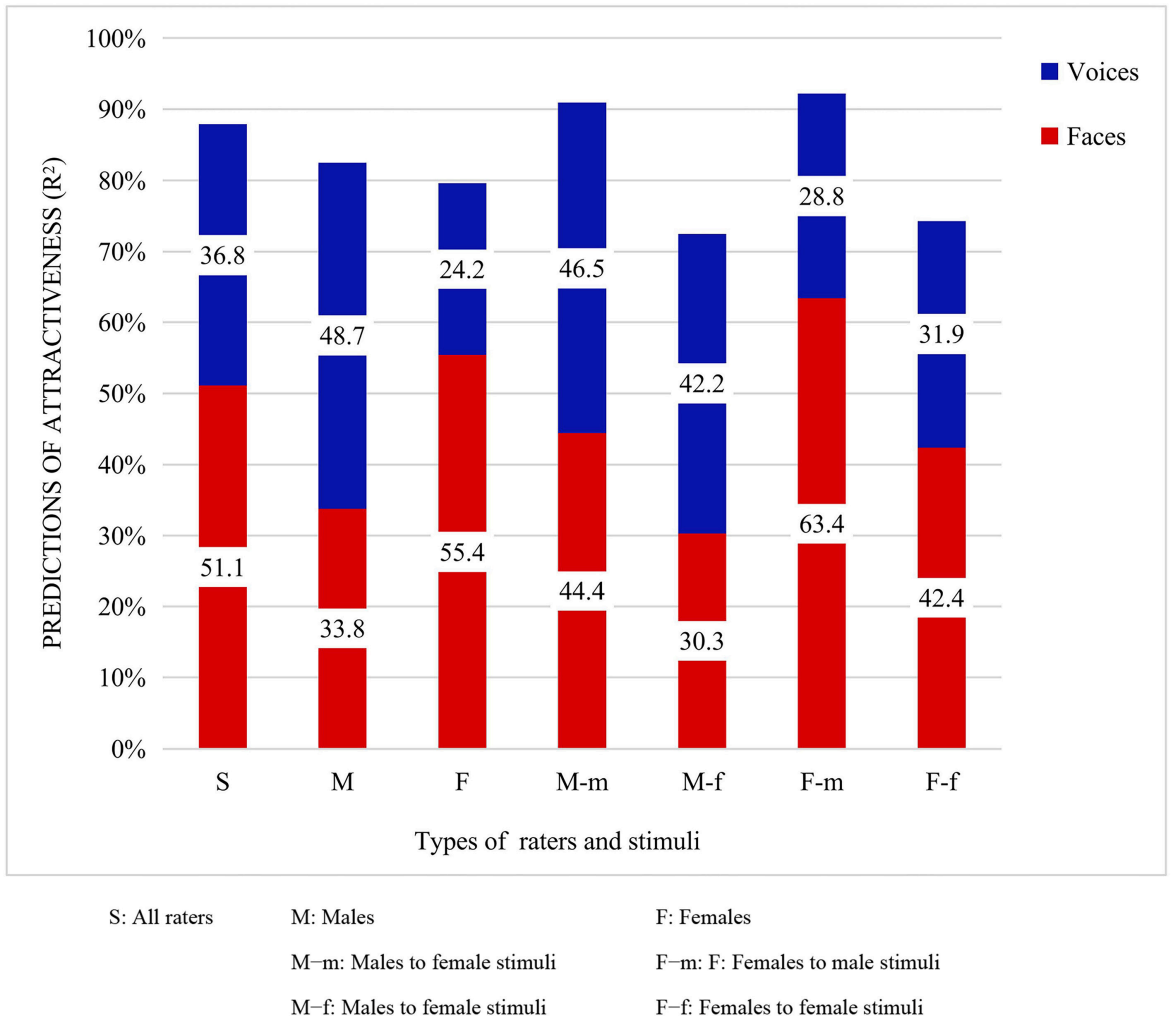
Regression analysis of predictions (predicted power R^2^) of FA and VA in multimodal stimuli in Study 2.

We employed multiple regressions to investigate the predictive effects of facial and vocal stimuli's attractiveness on multimodal stimuli's attractiveness (i.e., the weight of FA and VA to overall attractiveness). FA and VA in Study 1 were regarded as the nature of them, which were used to predict the perceived attractiveness score of multimodal stimuli in Study 2. Estimated regression coefficients (*R*^2^) were used to evaluate the extent of predictive effects of facial stimuli and vocal stimuli on multimodal stimuli. First, including all stimuli and raters regardless of their sex, the result showed that the predictive effects of facial stimuli (*R*^2^ = 51.5%) [*F*_(1, 76)_ = 79.41, *p* < 0.01] were higher than those of vocal stimuli (*R*^2^ = 36.8%) [*F*_(1, 75)_ = 228.40, *p* < 0.01]. Second, from further testing sex differences among raters, we found that among male raters, predictive effects of vocal stimuli were much higher than that of facial stimuli; whereas, among female raters, predictive effects of facial stimuli were higher than vocal stimuli. Third, we tested sex differences both in stimuli and raters. Specifically, we separately tested the predictive effects of male stimuli among male raters, female stimuli among male raters, male stimuli among female raters and female stimuli among female raters. Consistently, we found that among male raters, predictive effects of both male and female vocal stimuli were higher than those of facial stimuli; whereas, among female raters, predictive effects of both male and female facial stimuli were higher than those of vocal stimuli (detailed *R*^2^s see Figure 5 and Table 3). It is worth noting that the highest predictive effects appear at the evaluation of female raters toward male stimuli (63.4%).

**Table 3:** Estimated regression analyses of all raters, raters' sex and raters' sex × stimuli's sex (contribution rate *R*^2^).

	**Raters' sex**	**Stimuli's sex**	**Facial stimuli β**	***R*^**2**^(%)**	***F***	**Vocal stimuli β**	***R*^**2**^ (%)**	***F***
All raters			0.70^**^	51.5	79.41^**^	0.61^**^	36.8	228.40^**^
Raters' sex	Male		0.58^**^	33.8	144.87^**^	0.68^**^	48.7	72.22^**^
	Female		0.73^**^	55.4	94.47^**^	0.49^**^	24.2	89.05^**^
Raters' sex × stimuli's sex	Male	Male stimuli	0.66^**^	44.4	163.57^**^	0.70^**^	46.5	31.265^**^
		Female stimuli	0.55^**^	30.3	40.90^**^	0.62^**^	42.2	27.79^**^
	Female	Male stimuli	0.81^**^	63.4	62.45^**^	0.54^**^	28.8	128.94^**^
		Female stimuli	0.62^**^	42.4	31.47^**^	0.57^**^	31.9	44.42^**^

** *p < 0.01*.

Finally, we tested the difference between OMS (*M* = 5.05, *SD* = 0.93) and non-OMS (*M* = 4.99, *SD* = 0.93) with paired-sample *t*-test in order to investigate the OMS-facilitating effect. The result revealed that the difference between the attractiveness of OMS and non-OMS was significant [*t*_(111)_ = 2.82, *p* < 0.01, *d* = 0.07], indicating that the OMS-facilitating effect was significant to some extent.

### Discussion

As we found in Study 1, sex differences among raters were not found, suggesting that different-sex raters consistently evaluated FA, VA, and the overall attractiveness, which supported Hypothesis (2a). Moreover, we found EOSF among male raters rather than female raters, which also supported Hypothesis (2b and 2c) that only men would evaluate opposite-sex multimodal stimuli as more attractive than same-sex counterparts.

In multiple regression analysis, we employed estimated regression coefficients (*R*^2^) to indicate predictive effects of facial and vocal stimuli on multimodal stimuli (i.e., the weight of FA and VA on the overall attractiveness). For example, higher *R*^2^ of FA indicates that FA has a stronger impact on the overall attractiveness. FA, therefore, takes more weight in the process of perceiving and evaluating the multimodal stimuli's attractiveness. However, the weight of FA and VA shows some distinctions among different-sex raters—more weight of VA among male raters, but more weight of FA among female raters—probably because different-sex people are evolved to gain different (amount or importance) information from distinct physical cues.

Finally, the OMS-facilitating effect was found, indicating the higher attractiveness of OMS than that of non-OMS. OMS are more attractive than non-OMS may be because OMS are more harmonious than non-OMS, and the faces and voices in OMS are probably biologically associated with each other because both of them come from the same genes.

## General Discussion

In the current study, we did not find correlations between same-individual FA and VA. However, we found that different-sex participants consistently evaluated FA, VA, and overall attractiveness. Surprisingly, male raters evaluated female VA and overall attractiveness higher than female raters. This finding revealed that EOSF exists independently in VA. In general, FA predicted overall attractiveness more than VA, but the degrees of predictive effects of FA and VA show sex differences. In specific, FA predicted more than VA among women, while VA predicted more than FA among men. Finally, OMS-facilitating effect was found, illustrating that people evaluate OMS as more attractive than non-OMS.

### Correlations Between Same-Individual Physical Cues (FA and VA)

Focusing on the correlations between same-individual FA and VA, we did not find a significant effect on that, which is in line with a recent finding that facial and vocal attractiveness are uncorrelated (Zäske et al., 2018). In contrast, positive correlations between facial and vocal attractiveness were found by Valentova et al. (2017), however, only in women but not in men. Furthermore, although Hughes and Miller (2016) found that attractive/unattractive faces were associated with attractive/unattractive voices, this finding was based on people's “what-sounds-beautiful-looks-beautiful” stereotype, rather than the actual attractiveness. Thus, it remains unclear whether the evolutionary fitness distributes attractiveness (evolutionary superiority) to people's all or just some physical cues to make people superior in the mate processes. In practice, the attractiveness of a single physical signal can also benefit people, making them more appealing than others.

On the other hand, according to Rezlescu et al. (2015), the negative correlation between male FA and VA could be interpreted as “genetic compensation.” Given an amount of attractiveness an individual has possessed before birth, god either distributes more attractiveness to the face and relatively less to the voice, or more to the voice and relatively less to the face. In this theory, in order to possess a sufficient amount of evolutionary dominance, for example, the less-dominant FA needs a high-dominant VA to compensate, which results in a negative correlation between them. Therefore, although attractiveness of physical cues mainly depends on own “genes,” which are heritable (Barber, 1995), FA and VA may not have a robust or positive relationship.

### The Consistency of Evaluating the Attractiveness

The positive correlations of two evaluations on repeating stimuli suggest that people can accurately and consistently perceive and evaluate FA and VA with stable psychological criteria, although some people reported that they were hard to distinguish the VA from one and another due to their less perceived distinction. Based on that, we assume to some extent that the perception of VA may be implicit because participants can evaluate same VA twice in relatively consistent score even though they believe that they cannot distinguish them. However, the assumption has not been empirically tested in the present research.

We found that different-sex raters consistently evaluated FA, VA, and overall attractiveness both in Study 1 and Study 2. Raters, for example, were likely to highly evaluate attractive facial and vocal stimuli without significant sex differences, which is not like what people claim in our shared experience that men are not able to recognize the attractiveness of other men. In fact, they might be no less sensitive to perceive other men's attractiveness than women are. On the other hand, the EOSF was found only among male raters. Thus, EOSF may relate to the male strategy of short-term mate preferences, which developed over evolution (Buss, 2007). To be specific, men generally invest and take risks less than women in mate processes so that they tend to lower their standards as well as to increase their sensitivity and sexual interests toward female traits to get more contacts, sexual intercourse, and reproduction opportunities (Buss, 2007). Under this framework, men also need to evaluate same-sex attractiveness accurately since same-sex people are potential competitors. The necessity of accurately evaluating different-sex people can probably explain people's consistent evaluation of different-sex physical cues. Only if people can objectively and accurately evaluate different-sex FA and VA, can they motivate more social interests in attractive opposite-sex people and guard against attractive same-sex ones (Buss, 2007). However, EOSF was not found among female raters toward male stimuli probably because men value women's physical cues whereas women value men's resources, social status, and economic conditions (Buss, 2007; Tybur and Gangestad, 2011). Thus, women need to concern both physical attractiveness and resources among men, and it is unnecessary for women to overvalue male physical attractiveness.

### Weight of FA and VA in Overall Attractiveness

In general, FA has a more significant impact and takes more weight than VA in attractiveness evaluations. Rezlescu et al. (2015) supported that FA was valued more than VA when evaluating the attractiveness of the integration of impression (overall attractiveness in the current study). The distinction between FA and VA may be because people receive 80% information from environments through the visual channel (Zhu, 2000) and distribute more resources on visual senses. Previous researches proposed “Facial Overshadowing effect” (Cook and Wilding, 1997; Stevenage and Neil, 2014) when presenting facial and vocal stimuli together. In particular, the impact of vocal stimuli would become relatively weaker and impaired if they are presented alongside facial stimuli, and facial stimuli would remain dominant.

Then, we compared the weight of FA and VA on overall attractiveness in different sex. The results revealed different-sex preferences for different physical cues. To be specific, we found the dominance of FA among women, which also supported the “Facial Overshadowing effect.” FA takes more weight among women probably because women are less likely to be induced by EOSF than men. Thus, they directly evaluate the attractiveness through the visual channel in order to get more information about physical cues. However, we found the dominance of VA among men. As we found in Study 1, EOSF was only significant in VA rather than FA among male raters, suggesting that men value female VA more than FA to some extent. Theoretically, men might be able to gain more important or amount of information from female VA than FA. Nevertheless, it still needs further empirical research to investigate what factors influence male preferences for female VA over FA.

### OMS-Facilitating Effects

The OMS-facilitating effect was revealed by comparing OMS and non-OMS in Study 2, which means OMS were evaluated as more attractive than non-OMS. Previous evidence shows that people can correctly match which face and voice belong to the same person above the chance level (Mavica and Barenholtz, 2013), which supported our findings indirectly. OMS may improve the integrated impression or “first impression” as well as increase the perceived overall attractiveness since they are more harmonious than non-OMS. This finding not only in line with the previous study about effects of people's perception and recognition of OMS, but also revealed OMS-facilitating effect regarding attractiveness, which is more specific and practical for experimental psychology, especially for the field of sex robots and virtual sex.

### Implications to Sex Robots and Virtual Sex

Our findings could also contribute to the fields of sex robots and virtual sex in terms of their designs and popularization. Love and sex with robots (i.e., human-robot relationships) are being favored and accepted by human beings nowadays. This phenomenon is expected to be more common in the future—“love with robots will be as normal as love with other humans” (Levy, 2009).

From the perspective of evolutionary psychology, sex is designed for reproduction, and sexual passion and pleasure are “awards” of sexual behaviors ultimately. However, with human society developing, people's sexual needs have substantially exceeded their needs for generating offspring, which could be supported by the massive usage of contraceptives. Namely, a lot of sexual behaviors are not for reproduction any longer, but enjoyment instead. Unfortunately, some people engage in risky sexual behaviors to fulfill their sexual need, and some others (e.g., pedophile; Brents and Hausbeck, 2007) even commit sexual crimes for that. Given that, sex robots could be optimal alternatives for people to fulfill sexual needs with certain benefits suggested by Kolivand et al. (2017), involving preventing the dissemination of venereal diseases, reducing sexual crimes, and healing loneliness (especially for sexually vulnerable groups). Given these predictive and plausible benefits proposed by Kolivand and colleagues, what could sex differences in FA and VA found in our study probably contribute to the development and popularization of sex robots?

The current study has found that men prefer women's attractive physical appearance, especially preferring voices than faces; whereas women prefer men's faces in particular. Given this distinction, sex-robots designers could consider to particularly optimize voices of female robots due to the favor of men and to beautify the faces of male robots in order to win the affection of women.

Moreover, same-individual stimuli show facilitating effect (OMS-facilitating effect) on facial and vocal attractiveness, suggesting a plausible view to deal with the problem that sex robots are inadequately close to human beings (Rousi, 2017). Utilizing same-individual face and voice to design sex robots might be a feasible approach to make them more harmonious as well as to improve their overall attractiveness. Sex robots are more likely to be favored and embraced by people if they are designed to be more harmonious and humanoid. They are also more likely to become (short-term) sexual partners for human in order to reduce risky sexual behaviors, prevent sexual crimes, and heal loneliness.

### Limitations and Future Directions

Despite its strength, there are some limitations to the current study. Although both facial and vocal stimuli distributed normally, the limited number of facial and vocal stimuli may be the main reason why correlations between same-individual FA and VA were subtle. Future studies could collect a greater number of stimuli for more reliable and robust findings.

Another limitation is that we only employed a behavioral experiment to investigate different-sex evaluations of FA and VA. Future studies could test the activation level of brain areas with fMRI or ERP. It was reported that male nucleus accumbens, which is the pleasure center of the brain, become active when watching attractive female faces (Aharon et al., 2001). It suggests that fascinating opposite-sex faces are awards both on nerve and mentality for men.

Future studies could investigate whether different-sex people can gain different physical information from others, such as “evolutionary fitness,” “economic resources” or “fertility” (Wu et al., 2014). In this way, male preference for VA can probably be empirically explained instead of theoretically speculating.

Future studies could also investigate the impacts of some demographic information, such as health conditions, educational level and economic situations in people dating or mating, labels of which may mediate the effect of physical attractiveness.

## Conclusion

To conclude, people's ratings to attractiveness of faces, voices, and face-voice combinations are reliable and valid. Although people can consistently evaluate the attractiveness of physical cues (i.e., faces and voices in the current study) of different sexes, men still view vocal and overall attractiveness of women more attractive than those of men robustly. Same-individual FA-VA correlations were not found probably due to the limited amount of stimuli or the lack of effect actually. Faces generally play a more critical role than voices when evaluating the attractiveness, but the degrees of the weight of FA and VA on overall attractiveness indicate sex differences—men prefer voices and women prefer faces. Finally, OMS show an increased attractiveness, suggesting that people's preference and sensitivity to recognize same-individual combinations, which could contribute to the design and popularization of sex robots.

## Ethics Statement

The study was approved by the Human Research Ethics Committee of Anhui University. All participants gave consent to participate in the study and principles expressed in the Declaration of Helsinki were closely followed. Participants were undergraduate students. We did not obtain informed consent from the next of kin, caretakers, or guardians on behalf of the minors/ children enrolled in our study. Informed consent was obtained in written form from all participants.

Only one participant was 17 years old. We did not obtain consent from his guardians. This young college student was considered to have comparable intelligence and ability to adult students, and able to take charge of his behavior. According to the General principles of the Civil Law of the Peopleandamp's Republic of China; A minor aged 10 or over shall be a person with limited capacity for civil conduct and may engage in civil activities appropriate to his age and intellect; in other civic activities, he shall be represented by his agent ad litem or participate with the consent of his agent ad litem (Article 12, Chapter II). Therefore, we obtained the same consent from this participant as those above 18 years old, which was also approved by the Human Research Ethics Committee of Anhui University.

## Author Contributions

JH and ZY performed the experiments, analyzed the data, wrote the paper, revised each draft and did the final approval of the version.

### Conflict of Interest Statement

The authors declare that the research was conducted in the absence of any commercial or financial relationships that could be construed as a potential conflict of interest.
