# Supplementary material for: Sex Differences in Facial and Vocal Attractiveness Among College Students in China
Source: Front Psychol. 2019 May 22;10:1166. doi: 10.3389/fpsyg.2019.01166 (PMC6538682; doi:10.3389/fpsyg.2019.01166)
Supplement: Supplementary file 1 [file Table_1.DOCX]

**Correlation Coefficients of Materials (Reliability Analyzes)**

| Materials’ numbers | Facial materials (*r*) | Vocal materials (*r*) |
| --- | --- | --- |
| 1 | .86^**^ | .66^**^ |
| 2 | .85^**^ | .71^**^ |
| 3 | .83^**^ | .54^**^ |
| 4 | .80^**^ | .50^**^ |
| 5 | .76^**^ | .71^**^ |
| 6 | .72^**^ | .53^**^ |
| 7 | .80^**^ | .62^**^ |
| 8 | .71^**^ | .74^**^ |
| 9 | .87^**^ | .82^**^ |
| 10 | .69^**^ | .71^**^ |
| 11 | 70^**^ | .70^**^ |
| 12 | .86^**^ | .69^**^ |
| 13 | .75^**^ | .51^**^ |
| 14 | .87^**^ | .66^**^ |
| 15 | .75^**^ | .61^**^ |
| 16 | .69^**^ | .64^**^ |
| 17 | .71^**^ | .69^**^ |
| 18 | .78^**^ | .62^**^ |

*Note.*: ^**^. *p* < .01
