# Supplementary material for: Sex Differences in Facial and Vocal Attractiveness Among College Students in China
Source: Front Psychol. 2019 May 22;10:1166. doi: 10.3389/fpsyg.2019.01166 (PMC6538682; doi:10.3389/fpsyg.2019.01166)
Supplement: Supplementary file 2 [file Table_2.DOCX]

**Scores and Ranks of Attractiveness of Faces and Voices**

| Materials’ ranks | Facial numbers | Facial scores | Vocal numbers | Vocal scores |
| --- | --- | --- | --- | --- |
| 1 | 1 | 5.31 | 12 | 6.52 |
| 2 | 10 | 5.07 | 10 | 6.45 |
| 3 | 6 | 5.04 | 2 | 6.29 |
| 4 | 4 | 5.01 | 15 | 5.92 |
| 5 | 5 | 4.99 | 17 | 5.88 |
| 6 | 9 | 4.89 | 18 | 5.78 |
| 7 | 8 | 4.88 | 6 | 5.73 |
| 8 | 2 | 4.83 | 8 | 5.66 |
| 9 | 11 | 4.81 | 5 | 5.48 |
| 10 | 15 | 4.74 | 1 | 5.43 |
| 11 | 17 | 4.68 | 13 | 5.39 |
| 12 | 3 | 4.35 | 16 | 5.28 |
| 13 | 14 | 4.00 | 11 | 5.16 |
| 14 | 16 | 3.99 | 7 | 5.02 |
| 15 | 18 | 3.94 | 14 | 4.99 |
| 16 | 7 | 3.57 | 3 | 4.76 |
| 17 | 13 | 3.39 | 9 | 4.73 |
| 18 | 12 | 3.24 | 4 | 4.54 |

*Note.*: ^**^. *p* < .01
