# Supplementary material for: Sex Differences in Facial and Vocal Attractiveness Among College Students in China
Source: Front Psychol. 2019 May 22;10:1166. doi: 10.3389/fpsyg.2019.01166 (PMC6538682; doi:10.3389/fpsyg.2019.01166)
Supplement: Supplementary file 3 [file Table_3.DOCX]

**Correlation Coefficients of Combination Materials (Reliability Analyzes)**

| Materials’ numbers | Combination materials  （r） | Materials’ numbers | Combination materials  （r） | Materials’ numbers | Combination materials  （r） |
| --- | --- | --- | --- | --- | --- |
| 1 | .62^**^ | 27 | .64^**^ | 53 | .51^**^ |
| 2 | .72^**^ | 28 | .68^**^ | 54 | .55^**^ |
| 3 | .63^**^ | 29 | .57^**^ | 55 | .49^**^ |
| 4 | .52^**^ | 30 | .53^**^ | 56 | .53^**^ |
| 5 | .53^**^ | 31 | .53^**^ | 57 | .60^**^ |
| 6 | .64^**^ | 32 | .59^**^ | 58 | .55^**^ |
| 7 | .51^**^ | 33 | .37^**^ | 59 | .63^**^ |
| 8 | .49^**^ | 34 | .59^**^ | 60 | .54^**^ |
| 9 | .66^**^ | 35 | .62^**^ | 61 | .74^**^ |
| 10 | .59^**^ | 36 | .51^**^ | 62 | .50^**^ |
| 11 | .61^**^ | 37 | .54^**^ | 63 | .56^**^ |
| 12 | .71^**^ | 38 | .38^**^ | 64 | .61^**^ |
| 13 | .60^**^ | 39 | .51^**^ | 65 | .60^**^ |
| 14 | .74^**^ | 40 | .55^**^ | 66 | .53^**^ |
| 15 | .67^**^ | 41 | .61^**^ | 67 | .55^**^ |
| 16 | .69^**^ | 42 | .59^**^ | 68 | .53^**^ |
| 17 | .65^**^ | 43 | .58^**^ | 69 | .46^**^ |
| 18 | .46^**^ | 44 | .58^**^ | 70 | .54^**^ |
| 19 | .60^**^ | 45 | .53^**^ | 71 | .66^**^ |
| 20 | .44^**^ | 46 | .67^**^ | 72 | .40^**^ |
| 21 | .53^**^ | 47 | .68^**^ | 73 | .40^**^ |
| 22 | .58^**^ | 48 | .64^**^ | 74 | .51^**^ |
| 23 | .56^**^ | 49 | .66^**^ | 75 | .72^**^ |
| 24 | .53^**^ | 50 | .65^**^ | 76 | .63^**^ |
| 25 | .71^**^ | 51 | .64^**^ | 77 | .75^**^ |
| 26 | .61^**^ | 52 | .78^**^ | 78 | .73^**^ |

*Note.*: ^**^. *p* < .01
